# Concepts of healthy and environmentally sustainable diets clash with a life in transition – Findings from a qualitative study in urban Burkina Faso

**DOI:** 10.1080/16549716.2025.2457193

**Published:** 2025-02-12

**Authors:** Hannah Fülbert, Souleymane Zoromé, Roch Modeste Millogo, Ina Danquah, Alina Herrmann

**Affiliations:** a Heidelberg Institute of Global Health (HIGH), Heidelberg University Hospital and Medical Faculty, Heidelberg University, Heidelberg, Germany; b Institut Supérieur des Sciences de la Population (ISSP), Université Joseph Ki-Zerbo, Ouagadougou, Burkina Faso; c Center for Development Research (ZEF), Bonn University, Bonn, Germany

**Keywords:** Sub-Saharan Africa, nutrition transition, perceptions, health, sustainable nutrition, climate change, environment, diet, Burkina Faso

## Abstract

**Background:**

Sub-Saharan African countries like Burkina Faso face a dietary transition and are experiencing a shift in disease burden.

**Objective:**

We explored perceptions of healthy and environmentally sustainable dietary habits in urban Burkina Faso in order to tailor nutritional interventions to the local population and ultimately improve public and planetary health.

**Methods:**

We conducted an exploratory qualitative study with semi-structured face-to-face interviews in three informal and two formal neighborhoods of Ouagadougou. The sample comprised 36 adult participants. The interviews were conducted in Mooré and French, audio-recorded, and transcribed verbatim. Data were analysed inductively, using thematic analysis.

**Results:**

Participants described their ideal healthy and environmentally sustainable diet as traditional, local, natural, pure, organic, and transparent in terms of food production, processing, and preparation. Perceived barriers to achieve such diets were: limited financial resources, reduced availability of products and limited time for food preparation. Furthermore, participants highlighted discordant food preferences in the family, and a lack of understanding around the interconnection between nutrition, health and the environment as barriers. Most of these barriers were aggravated by the experience of a life in transition due to modernizing lifestyles, globalizing food systems, and a changing environment.

**Conclusions:**

Participants’ ideal of a healthy and environmentally sustainable diet clashed with a life in transition. To improve public and planetary health, interventions should aim to empower individuals, alleviate financial constraints, and shape global and local food environments.

## Paper concept

- **Main findings:** Our participants had clear conceptions regarding healthy and environmentally sustainable diets, yet, the ideal often differed from reality, mainly due to economic barriers.
- **Added knowledge**: Unlike quantitative studies that place Burkina Faso at the early stages of the nutrition transition, our participants shared perspectives on changing dietary patterns going along with lifestyle, food system and environmental changes.
- **Global health impact for policy and action**: Improving access to healthy and environmentally sustainable diets requires multilevel strategies, including information campaigns, targeted subsidies and taxes, revised national dietary guidelines, and concepts to make local agriculture more climate-resilient.

## Background

Since the 1970s, global diets have drastically changed [1]. This so-called nutrition transition is characterized by a shift from traditional to ‘modernized’ diets [2]. The latter are characterized by higher consumption of salt, saturated fats, simple carbohydrates, and calories, as well as lower intakes of whole-grain cereals and products high in fibre [1,3,4]. As a result, overweight and obesity prevalence, as well as diet-related diseases are increasing globally, having a negative impact on life expectancy [5]. In sub-Saharan Africa (SSA), half of the countries are currently in the early stages of the nutrition transition. Meaning that the amounts of consumed fats and processed foods are still rather low in comparison to Europe and the Americas [2]. However, diets are rapidly changing on this subcontinent [1,4,6]. These dietary shifts combined with high urbanization rates, a sedentary lifestyle [2,7], and aging populations promote the double burden of disease compounding infectious and non-communicable diseases (NCDs) in SSA [4,8].

To date, Burkina Faso is in the early stages of the nutrition transition [9]. Yet, the number of individuals with overweight and obesity has risen notably [10]. The burden of NCDs is estimated to overtake that of communicable, maternal, neonatal, and nutritional diseases by 2035 [9]. This shift is most notable in capitals such as Ouagadougou [11–13] and urban areas of West Africa. They are experiencing rapid increases in the consumption of animal-based and ultra-processed products [14]. Combined with increasingly sedentary lifestyles, this contributes to a rising overweight and obesity prevalence in Ouagadougou [13]. Currently, 28.4% of the adult population aged ≥35 years are overweight [15], and levels of overweight and obesity prevalence among urban women are among the highest compared to other urban SSA regions [16]. At the same time, disease burden from undernutrition and micronutrient deficiencies is still high in Ouagadougou, resulting in the double burden of malnutrition [11,17].

In addition to the described health impacts, the current global food system immensely contributes to greenhouse gas emission, threatens biodiversity, and increases freshwater withdrawal [18]. In order to attenuate the ongoing environmental crisis, it is important to target dietary practices [19,20]. Recommendations for diets with low environmental impact often coincide with guidelines for healthy diets [18]. One example is the planetary health diet, which provides a reference diet to feed a global population of 10 billion people in 2050 within planetary boundaries [20].

Thus, for countries like Burkina Faso, the implementation of a culturally adapted planetary health diet [20] might be a powerful tool for the prevention of overweight and obesity [21], and could simultaneously address insufficient dietary diversity and undernutrition [5]. In order to tailor dietary interventions to the need of Burkinabé people, knowledge about current dietary practices and beliefs around healthy and environmentally sustainable diets is essential. Currently, there is a lack of empirical evidence on beliefs around healthy and environmentally sustainable diets in low- and middle-income countries [22–25] and on the factors that influence dietary habits in urban Africa in general [26]. Understanding the latter is crucial for identifying preventive measures, such as dietary and lifestyle interventions [27]. This study aimed to explore urban dwellers’ perceptions of healthy and environmentally sustainable diets in Ouagadougou and barriers to their implementation. The purpose is to inform the development and implementation of dietary interventions, which aim to improve the healthfulness and environmental sustainability of diets in SSA.

## Methods

### Study design and setting

**Figure 1.**
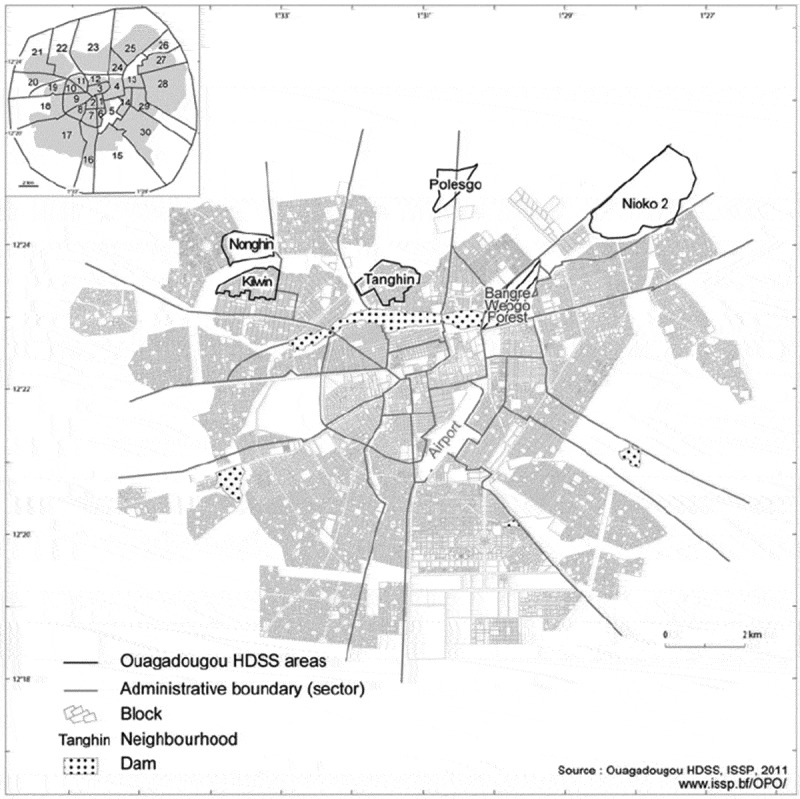
Five settlements in the north of the capital covered by the HDSS Ouagadougou. Nonghin, Polesgo, and Nioko-2 in the most peripheral areas of the city represent settlements of unplanned growth or so-called informal settlements; Kilwin and Tanghin represent the city’s formal settlements [29].

This exploratory qualitative study constitutes the formative component of a larger research project on sustainable diets in SSA, which implemented a dietary intervention considering aspects of a planetary health diet [28]. This qualitative study was conducted within the area of the Health and Demographic Surveillance System (HDSS) Ouagadougou. HDSS Ouagadougou is the first urban demographic surveillance system in West Africa and covers a population of about 82,000 inhabitants [29]. It spans over five districts of Ouagadougou (see Figure 1), two official districts within the city (formal settlements; Kilwin and Tanghin) and three unregulated districts at the periphery of the city (informal settlements; Nonghin, Polesgo and Nioko 2). Ouagadougou is one of the fastest growing urban areas in West Africa [16,29], and is characterized by high prevalence of overweight, obesity, and NCDs [15,16]. The Human Development Index of Burkina Faso is 0.44 (UN 2022), putting the country in 196th place out of 204 [30].

### Sampling and recruitment

To capture multiple perspectives, purposeful maximum variation sampling was applied [31]. We sought for maximum variation in age, sex, and settlement type (formal and informal settlements) and stratified for the latter two sampling criteria. Only adult participants, aged 18 or older, were included. Individuals fulfilling the sampling criteria were identified via the HDSS database and approached via telephone. Interested individuals chose the time, location, and language (Mooré or French) of their interview. Participation in this study was voluntary, interviews were only conducted after informed consent was signed, and local COVID-19 hygiene measures at the time were respected (also see ethics statement at the end of the manuscript). The final sample size was defined by the principle of data saturation [31].

### Development of the qualitative interview guide

A semi-structured interview guide was developed based on the research objectives by the authors. This interview guide allowed for a natural conversation, while giving enough space to talk about pre-defined topics to answer the research questions [32]. An English version was developed, which was subsequently translated into French and the local language Mooré with the local researchers. The interview guide consisted of eight sections (see Table 1). In this paper, we present the findings from sections number 1 to 4, 6 and 8 (six out of eight sections). This is because the interview guide also covered questions about the perceptions of body shape (section 5) and a weight-loss intervention (section 7), which do not answer the research questions of this paper and will be published elsewhere.

**Table 1.** Outline and content of the semi-structured interview guide.

No.	Section	Content
**1**	Introduction	Presentation of the interviewer, brief explanation of the research subject and the objectives of the interview, information on data use and privacy issues, confirmation of informed consent, room for questions by the interviewee before the start of the interview, encouragement of the participant to speak freely
**2**	Warm-up questions	Reflections on a typical everyday diet and factors influencing dietary choices, perception of the meaning of nutrition, thoughts of most important aspects while eating
**3**	Perceptions of current and traditional dietary habits	Reflection on preferred dishes and drinks, as well as dishes and drinks the interviewee does not like, questions related to food purchase and food origin, perception of changes between today’s nutrition and a typical diet of the past, questions about traditional food, experiences related to eating food outside the home, reflections on the role of money in everyday diets
**4**	Perceptions of healthy and sustainable diets	Perceptions of food and diets considered as healthy or unhealthy, perceptions of the environmental impact of food, questions about climate change, reflections on a healthy everyday diet that respects the environment
**5**	Body shape perception	Perceptions and associations related to a slim or big body shape, questions about good and bad health consequences of overweight, perception of public health implications related to overweight in Ouagadougou and Burkina Faso
**6**	Changing one’s dietary habits	Questions about desired dietary changes, reflections on promoting and hindering factors
**7**	Acceptability of a dietary weight-loss intervention	Questions about the necessity of a dietary weight-loss intervention in the context of Ouagadougou, brief information on the different elements of the planned intervention, questions regarding first impressions, comprehensibility, and more or less interesting elements, reflections on the program’s objectives and required effort, specific questions on some details like a possible cooking session or physical activity during the program
**8**	Conclusion	Review of the interview and particularly interesting topics, possibility to add any additional thoughts and perceptions, appreciation for participation

### Data collection

Data collection took place from February to March 2021 in Ouagadougou, Burkina Faso, and was performed by three local interviewers. Their training before data collection covered theory on qualitative research epistemology, as well as a practical session on qualitative interviewing. One local scientist with background in qualitative research supervised the data collection and debriefed daily with the interviewers (SZ). The research teams from Burkina Faso and Germany met online bi-weekly to discuss difficulties and observations. After about one-third of the interviews, the field supervisor and the researchers from Germany undertook an in-depth debriefing (SZ, AH, HF).

The interviews were conducted in French (*n* = 18) or Mooré (*n* = 18), according to the participants’ preference, and were audio-recorded on site.

### Data management and analysis

The interview duration ranged between 28 and 130 minutes, the mean was 50 minutes and the median 47 minutes. All interviews, including those in Mooré, were transcribed verbatim into French and pseudonymized.

We employed thematic analysis according to Braun and Clarke [33], following a fully inductive approach [34]. The software NVivo 12 Pro (Alfasoft GmbH, Germany) was used to support the analysis process.

In the first step, the principal analyst (HF) listened to the audio files, read the transcripts and took reflective notes for familiarization with the data. In the second step, she coded the data and held bi-weekly meetings with an experienced qualitative researcher (AH) to reflect on the coding scheme and the emerging themes and sub-themes. Due to technical reasons, the qualitative researcher from Ouagadougou (SZ) shared his feedback in written form. In the third step, HF and AH developed themes and sub-themes at higher abstraction level. Apart from NVivo, HF created graphic illustrations to visualize and facilitate the analysis process. In the fourth step, distinctions between the themes were made clear, every theme’s grounding in the data was checked by re-reading all references, and each theme was clearly defined and named. In the fifth and last step, the principal analyst (HF) wrote a detailed summary in close consultation with the research team.

### Reflexivity statement

Qualitative research is never uncoupled from the researchers’ backgrounds, perspectives, and personal beliefs [35]. Here we report on the researchers’ familiarity with the research context, as we have identified this aspect to be most relevant to our study.

Principal analyst HF is a white female medical doctor from Germany (student at the time of data collection and analysis), who is fluent in French. Due to the COVID-19 pandemic and the related travel restriction during data acquisition, she could not collect data herself. The field supervisor (SZ) and the three interviewers were Burkinabé staff of a renown local University (University Joseph Ki-Zerbo). They are trusted community members with a lived experience in the study context. To critically reflect on her interpretation of the data, HF gathered information about Burkina Faso through literature and regular exchange with the research team on site.

## Results

The final sample comprised 36 individuals (18 men, 18 women). Seventeen participants resided in the formal settlements of Kilwin and Tanghin, and 19 respondents resided in the informal settlements of Nonghin, Polesgo, and Nioko-2. The mean age of the participants was 42 years, with a range from 22 to 73 years.

**Figure 2.**
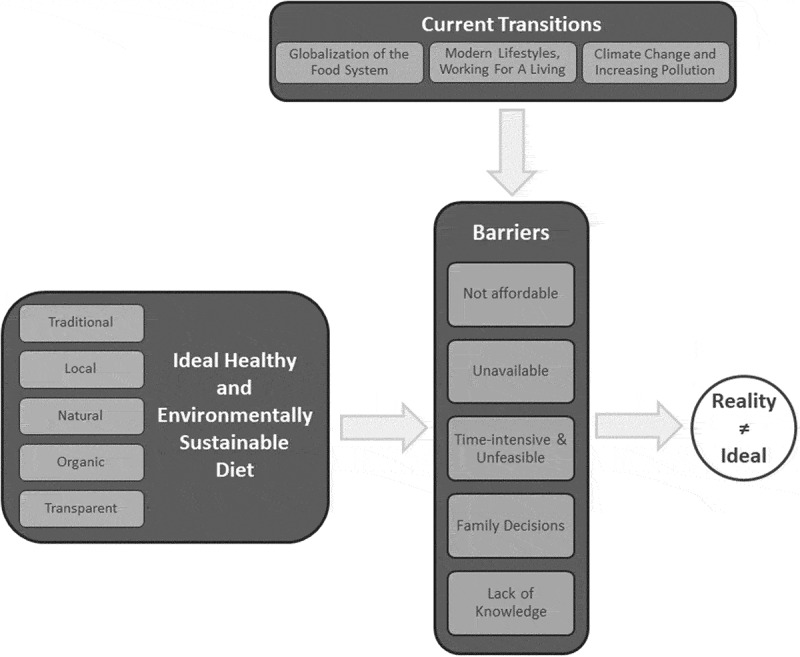
Illustration of overarching theme (white circle), main themes (dark grey boxes), categories (light grey boxes), and their relations (indicated by arrows) revealed by the qualitative analysis. For further details, see additional file 1.

The identified overarching theme was ‘concepts of healthy and environmentally sustainable diets clash with a life in transition’, shown as a circle in Figure 2. The three main themes underpinning this are (see Figure 2): an ‘ideal healthy and environmentally sustainable diet’ (theme 1), ‘barriers’ (theme 2) in attaining this ideal diet, and ‘current transitions’ (theme 3) on the levels of the food system, lifestyle, and the environment complicating these barriers

### Theme 1: ideal healthy and environmentally sustainable diet

Our participants stressed the importance of their diet’s healthfulness and often brought up the topic by themselves. If not, probing questions revealed detailed insights into their understanding of healthy diets. The environmental sustainability of diets was rarely introduced by the participants, but needed to be frequently probed by the interviewers. Some respondents saw a broad interconnection between health and environmental sustainability: ‘*If it is not good for the human being, it is not good for the earth either'. (P23, f, if, 50 years).* (Pseudonymized participant code: P n = participant number / f = female, m = male / fo = formal neighbourhood, if = informal neigbourhood / age at the time when the interview was conducted).

The qualitative analysis revealed five characteristics of an ideal healthy and environmentally sustainable diet: Food should be (i) traditional, (ii) local, (iii) natural, (iv) organic, and (v) transparent according to the participants. There was some overlap across these categories, for instance local food was often related to transparency. Table 2 summarizes the categories, codes and illustrative quotes. At the end of this section, we illustrate additional characteristics attributed exclusively to healthy diets.

**Table 2.** Categories of theme 1: characteristics of an ideal healthy and environmentally sustainable diet.

Category	Codes	Illustrative quote
Traditional	Grandparents ate primarily home-made and home-grown food	*“Our grandparents consumed what they grew. They knew where their products came from and even the sauce. What the women used was what they grew. So everything was truly natural. […]The quality of the sauces is different as well. The grandparents made their sauces and crushed their peanuts to make peanut paste, but today we buy everything” (P28, m, fo, 41 years)*
	Grandparents ate more simple and natural	*“I tell myself that our grandparents, their food was much healthier, it was prepared in a simpler way with the soumbala that they gained and apart from that, it was good quality soumbala” (P22, m, fo, 33 years)*
	Grandparents ate less varied food	*“For me, the difference is that in our grandparents’ time, there was no variety of dishes. It was either tô or beans, something like that. But nowadays, we can make a lot of dishes, so we can vary. […] They didn’t have a choice.” (P15, f, if, 30 years)*
	Traditional recipes	*“I think that well-prepared traditional food is healthy and sustainable.” (P28, m, fo, 41 years)*
Local	Authentic	*“But if you know that the rice you bought was grown in that village and then you can confirm its entire process up to the point where it comes to you, well then we would say that it’s authentic and that it’s fine.” (P28, m, fo, 41 years)*
	Local food healthy and sustainable	*“What I was going to say here, what I consume, the rice that we produce here is really healthy and sustainable. I like it and I know that it doesn’t inflict anything bad like that!” (P3, m, fo, 60 years)*
Natural	Chemicals associated with growing or preserving food environmentally unsustainable	*“[…] there are pesticides and so on that we use to produce our food. But these pesticides and others also pollute the soil. So that can drive climate change.” (P28, m, fo, 41 years)*
	Processed food environmentally unsustainable	*“[…] there are foods that are processed in factories. Machines are used that pollute the air. [...] For me [a healthy diet that respects the environment] is to insist on natural products. Products that have not gone through factories” (P15, f, if, 30 years)*
Organic	Organic healthy environmentally sustainable	*“The family, if you feed your family properly, it’s local and organic rice, that’s healthy.” (P3, m, fo, 60 years)*
	Organic healthy environmentally sustainable	*“Otherwise, if you eat mainly organic food, I think that this can solve the phenomenon [of climate change] a bit.” (P28, m, fo, 41 years)*
Transparent	Importance of knowing ingredients, conditions, and product origin	*Unhealthy food is the consumption of products of which you really don’t know where they come from and under what conditions they were made; that’s what I call unhealthy products.” (P22, m, fo, 33 years).*

#### Traditional

Many participants perceived their grandparents as healthy and strong which was frequently attributed to their dietary habits. The participants described that their grandparents cultivated, processed, and prepared the food they needed themselves and to a high quality. This was also perceived as beneficial for the environment. Knowing the conditions along the food chain and being in control of them was evaluated as positive by the respondents. When asked to provide examples of healthy foods, participants named traditional dishes mainly based on cereals, beans, leaves, and legumes. However, limited diversity of traditional diets was also seen as affecting health negatively.

#### Local

Participants rated local food as trustworthy and authentic, as they could better reproduce its provenance. Local cultivation practices were described as environmentally sustainable, organic and associated with short transportation, which was seen as desirable. The availability of local products was perceived as highest on local markets.

#### Natural and pure

Products which do not contain chemicals, pesticides, fertilizers, genetically modified organisms, or preservatives were perceived as ‘natural and pure’. This was explained by the assumption that chemicals could negatively affect soil, water, climate, insects, and human health. Preservative use was mainly connected to imported food.

#### Organic

Organic agriculture was related to benefits for humans and the environment, including climate change. Therefore, it was described as preferable over other cultivation practices by some participants.

#### Transparent

Individual control over and knowledge about production, processing, and preparation conditions was perceived as crucial to evaluate a diets’ healthfulness. Therefore, a diet was more likely assessed as healthy, environmentally sustainable, or desirable by the participants, when they felt they could verify and judge the conditions along the food chain.

#### Further features of healthy diets

Healthy food alone was associated with hygiene, homemade preparation, food variety, quality and simplicity. Cooking food thoroughly and food hygiene were described as contributing to a diets’ healthfulness. Food hygiene comprised hand washing, food cleaning, and a clean cooking environment. According to several participants, such principles can be best adhered to at home. Because of higher confidence in one’s own cooking skills and control over the ingredient quality, homemade food was cited as preferable over foods purchased or consumed elsewhere. Moderate use of salt, fat, and sugar was also emphasized by some participants. Finally, food variety was described as crucial to provide the body with enough nutrients and vitamins: *‘We would like to vary our dishes for example. Eating rice at noon, tô in the evening and why not yam the next day, that would be a lot of vitamins. Otherwise, if we content ourselves with the tô, the body does not receive any vitamins. The tô contains nothing.’ (P14, f, fo, 50 years).*

### Theme 2: barriers

Five barriers to adhere to healthy and environmentally sustainable diets emerged from the participants’ answers, as depicted in Table 3: (i) Financial situation, (ii) availability and offer, (iii) time and feasibility, (iv) family decisions, and (v) lack of knowledge.

**Table 3.** Illustrative quotes on experienced barriers to adhere to healthy and environmentally sustainable diets.

Category	Exemplary codes	*Illustrative quotes*
Financial resources	Inaccessibility of healthy, organic food and balanced diets without money	*“[…] we are in a world that is lost, to eat healthy you really have to have the means. If you don’t have the means you can’t eat well. That’s it.” (P18, m, if, 55 years)*
Availability and offer	Locally cultivated food seen as insufficient in amount and offer	*“[…] you go in these stores and look at the different brands of rice, this is an example, you will see that there is no local rice. It is difficult to get local rice […] So that’s an obstacle. […] In addition to that, I tell myself that imported rice is much cheaper than local rice, so these are difficulties.” (P22, m, fo, 33 years)*
Time and feasibility	Ready-to-serve meals seen as affordable and easy to prepare but non-transparent	*“But now, cooking at home, it’s quite expensive. What is already prepared, you don’t waste time, and it doesn’t require too many means either.” (P13, m, if, 22 years*
Family decisions	Influence of family members on dietary practices	*“The children can influence the decision. If you are going to cook and then the child doesn’t eat, or your husband doesn’t like the meal, you will do what the family wants. […] what you want doesn’t count, yes, you cook for the others!” (P7, f, fo, 31 years)*
Lack of knowledge	Knowledge as precondition for beneficial dietary behaviour	*“I have just realized that in Burkina, even I, we don’t have an idea about nutrition. What are the foods that can bring health to man, what are the foods that man must consume to have vitamins? Most do not know this. […] We don’t have an idea which foods can bring what, which foods provide vitamins. Well, some rare times they show it on TV, but we don’t have structures that can advise people to have healthy food. So that’s why ignorance can also make many people consume food believing that it is good food when in fact it is food that will kill them slowly.” (P34, m, if, 49 years)*

#### Financial situation

Money and its relation to an individual’s diet were thematized extensively by the participants and seemed to represent the most important barrier. Frequently, money was portrayed as a determinant of daily dietary choices and even equated with access to food. Hence, in times of financial constraints, the participants described to adapt their diets according to available means. In our sample, hunger was described as a recurrent issue among many households. As a result of limited choices, healthfulness and food quality were described to become secondary. Nourishing and satisfying the whole family with whatever food is available was the highest priority, also pushing considerations of taste and individual preferences into the background. Perceived higher costs of healthy products, like fruits, vegetables, and local dishes, were described to further complicate the implementation of healthy and environmentally sustainable diets. The current importance of money for daily nutrition was rated as higher in the city than in rural areas.

#### Availability and offer

The participants felt that healthy food is increasingly difficult to obtain. This was also the case for local and organic products. Consequently, evermore imported foods are purchased, even though they were often perceived as dubious and less healthy but cheaper alternatives. Furthermore, the availability of food at the preferred location for food purchase, mostly at nearby markets or supermarkets, was mentioned as an influencing factor of dietary choices.

#### Time and feasibility

On weekdays, the time required for preparing and eating desirable food versus the available time did not correspond according to the participants. Therefore, many ate at their workplace and bought food in canteens or at kiosks instead of returning home for lunch. This was often negatively perceived, because the participants felt that they had to eat dishes they did not like and/or consider unhealthy. After work or school, particularly when nobody cooked at home, preparing something quick, like noodles or ready-to-serve meals, was preferred over cooking a time-intense meal, such as a traditional dish.

#### Family decisions

Family members and other peers were named as complicating adherence to healthy and environmentally sustainable diets. In some cases, the family head (‘chef de famille’) was seen as deciding on the family’s nutrition, limiting individual freedom of choice. Furthermore, children were highlighted as influencing the family’s diet by requesting certain products and rejecting others.

#### Lack of knowledge

Not knowing about a beneficial, balanced diet was described as a barrier to follow a healthy and environmentally sustainable diet. Therefore, many participants wished to learn about the relations between nutrition, health, and the environment to make more conscious choices. To achieve this, one respondent highlighted the need for appropriate structures to provide trustworthy nutritional advice and recommendations to the population.

### Theme 3: current transitions

Throughout the interviews, various transitions were portrayed by the participants. These shifts were said to influence health and environmental issues and people’s diets. Participants experienced changes mainly in regards to the food system, lifestyles, and the environment, as illustrated in Table 4.

**Table 4.** Illustrative quotes on theme 3: current transitions at the food system, lifestyle, and environmental levels.

Sub-category	Codes	Illustrative quotes
Globalization of the food system	Many changes and differences in today’s nutritional behaviour compared to the past	*“There is a big difference […] eating habits have changed a lot. In the past, it was a lot more tô made from millet, corn. Today it’s much more rice and pasta. So it’s not the same thing.” (P28, m, fo, 41 years)*
	Modern and modernized food replacing the traditional	*“Traditional dishes nowadays have become modern. […] we have made them modern […] Nowadays, if we want to prepare beans we prepare it with condiments, we make a sauce, we add Maggi. It’s no longer beans, it becomes something else.” (P1, m, fo, 38 years)*
	Changing production conditions: Presence of chemical products, fertilizers, and genetically modified organism (GMO) in today’s food	*“[…] fertiliser like that, chemicals; today it’s just that […] And things have changed because in the past we used to eat without worries because the meals were not toxic. There were no chemicals. But today we only eat chemicals.” (P3, m, fo, 60 years)*
	Globalized world promoting food import	*“[…] Europe was not as close as it is today. From one day to the next rice can leave America and land here, whereas our grandparents did not know that.” (P34, m, if, 49 years)*
	Today’s nutrition seen as unhealthy, problematic, toxic, inferior in quality and non-transparent	*“[…] people have adopted what is modern at the expense of the nutrition of our grandparents. And that’s why diseases are increasing. The fact that we are abandoning our grandparents’ diet in favour of the modern one is why we are exposed to more diseases.” (P23, f, if, 50 years)*
	Children preferring rice over tô	*“Our children spend their day hungry because they don’t like tô. They are ready to go to school hungry because of the tô. But when we prepare rice, they are very happy. But when it’s tô, they don’t eat it. They are even ready to sleep hungry.” (P14, f, fo, 50 years)*
Modern lifestyles, Working for a living	Time and feasibility increasingly important when preparing food	*“People spend less time cooking than they used to. Today, for example, women, because they work, are not always at home to take their time to prepare food, so everyone is looking to prepare something quick. This leads to the use of manufactured products that allow you to do things quickly and I think that this plays a lot on food choices.” (P16, m, fo, 48 years)*
Environmental Changes	Climate Change	*“[…] with the increase in temperature from March, there is less water. So the market gardeners can’t continue to produce in their gardens. […] So the prices will quickly increase […] you can’t eat fresh products anymore. This is the link to climate change. Also, if the agricultural season has been very bad due to irregular rainfall, it will mean that we won’t have enough maize or millet on the market, and so we will have a price problem, and if the prices are too high we will be obliged to reduce the quantities that we eat. Speaking of the temperature too, […] because it is much hotter, the demand for water is also increasing.” (P16, m, fo, 48 years)*
	Pollution	*“If we see the proliferation of bags, and even bottles that we throw away, all that, it has an impact […] these plastic bags that really degrade our environment, that’s it and the pollution […] all that has negative effects […] it’s degradation, that means that it even destroys the soil.” (P34, m, if, 49 years)*

#### Globalization of the food system

In the past, people were described as being more closely connected to food production and processing. Nowadays, few still cultivate their own food, according to participants. Instead, the availability of imported and modern foods in supermarkets is increasing. However, descriptions of changing conditions along the food chain were related to more than just imported foods. In fact, local farmers were said to abandon environmentally sustainable practices in favor of chemicals, fertilizers, pesticides, and genetically modified crops. This development was viewed critical for health and the environment, soil and water pollution.

Moreover, the described changes in food supply and availability in the urban setting were said to come at the expense of local and traditional dishes, which were perceived to decline. Cheaper prices of imported goods, particularly regarding rice, were mentioned as another factor contributing to the increasing popularity of novel products. However, imports were connected to higher food variety throughout the year, which was perceived as beneficial. The participants highlighted this aspect when comparing themselves to rural dwellers.

The parents’ and grandparents’ generation often perceived recent dietary changes as negative and unhealthy. In contrast, the younger generation seemed more open to new products, and increasingly hostile to traditional dietary habits. In particular, it was described that the traditional cereal-based staple food tô is now often replaced by rice, frequently due to children’s preferences. Yet, some participants also expressed curiosity towards novel products.

Finally, today’s dishes were perceived as containing more additives, such as ‘chemicals’ or fat, salt, and sugar, compared to rather simple and pure traditional dishes. The participants felt this resulted in traditional recipes being modernized and modified.

#### Modern lifestyles and working for a living

According to the respondents, it was sometimes unavoidable to consume dishes perceived as unhealthy, especially when they had to eat out in canteens or at kiosks during work. This was another lifestyle change from the past, when subsistence farming was more common. Moreover, time spent on cooking was described as decreasing recently. The participants linked this to a growing number of women in the workforce. This was said to result in an increasing popularity of manufactured, quick, or ready-to-serve dishes. The latter were additionally perceived as low-cost.

#### Environmental changes

Environmental change, mainly in terms of climate change, was portrayed as an amplifier for health risks and food insecurity. The latter was explained by increased prices of local food products due to rain irregularities and hotter temperatures, while imported and modern foods were perceived to remain affordable. Such conclusions were mostly based on personal observations of changing climatic and environmental conditions: *‘It doesn’t rain enough anymore, the heat is much stronger than before’* (P2, f, fo, 53 years).

Furthermore, environmental degradation, especially soil degradation, was linked to food and nutrition by our participants. Particularly, a causal relation between the inconsiderate throwing away of plastics and soil degradation was emphasized.

As a result of a transitioning food system, lifestyle, and environment, diseases were seen to multiply, and contemporary people were perceived as less strong and less healthy than the grandparents or people living in rural regions. New, often inexplicable diseases observed by the participants, which they related to food, constituted hypertension, type 2 diabetes, and cancers. An increase in sedentary activities in the city was said to further exacerbate current health issues, mainly related to overweight. This development was of great concern because health was considered the highest possible good: *‘In everything it is health that counts. If you are healthy, the rest you can do'. (P25, f, fo, 42 years)*.

### Overarching theme: reality clashes with the ideal healthy and environmentally sustainable diet

Most participants had very clear ideas about healthy and environmentally sustainable diets which they would like to implement in their household. However, due to complex barriers amplified by recent transitions at larger levels, dietary choices were described as unplanned and limited, and the ideal was seen as frequently unattainable.

Thus, instead of following the ideal, actual diets were guided by the aim to feed all family members with available resources. Concretely, imported products were described as increasingly replacing the local ones. Furthermore, due to urban lifestyles, time for food preparation at home was limited, and thus, eating outside of the home was portrayed as becoming increasingly popular. For many respondents, this experienced reality stood for unhealthiness, harms for the environment, limited personal control, and a poor diet, resulting in frustration.

## Discussion

### Summary of main findings

The participants had clear ideas and preferences regarding healthy and environmentally sustainable diets, yet, numerous barriers, most notably economic ones, complicate adherence to the ideal. Environmental changes, as well as comprehensive transitions in the food system and people’s lifestyles, such as working for a living, were identified as contributing to these barriers.

### Concepts of healthy and environmentally sustainable diets clash with reality

How people define healthy eating depends on their socio-cultural context, resources, personal ideals, experiences, and knowledge [24,36]. Still, several broader aspects seem to be repeatedly attributed to healthy eating across different countries and settings [24]. Regarding healthy and sustainable eating, the literature found that people more easily characterize healthy diets than sustainable diets [23,37]. This aligns with our impression that more prompting was required in our sample to gain insights into perceptions on environmentally sustainable diets.

Most of the characteristics of healthy and environmentally sustainable diets mentioned by our participants were related to the conditions along the food chain, particularly production, processing, and preparation manners. The main attributes ‘traditional’, ‘local’, ‘natural and pure’, ‘organic’, and ‘transparent’ were associated with high food quality. These aspects have been associated with perceptions of healthy and sustainable diets in other contexts [22–24,36,38,39].

Consistent with a qualitative study on pregnant women from SSA residing in the United States [39], urban adults in our sample further emphasized the importance of low pesticide and preservative concentrations in their diet, and their preference for organic products. In accordance with existing evidence [22,24], our findings also revealed the importance of food hygiene for this urban Burkinabé sample. This aspect was particularly highlighted when comparing home-made food to dishes offered elsewhere, corroborated by Paquette [24].

Fruits and vegetables are frequently associated with healthy diets in other contexts [24,36]. Furthermore, a high proportion of fruits and vegetables is the main component of many theoretical concepts for a healthy and sustainable diet, such as the EAT-Lancet Planetary Health Diet (PHD) [20]. In this study, fruits and vegetables were enumerated as healthy foods, yet, they were not described as main components of a healthy and sustainable diet. They were rather portrayed as expensive and at times unaffordable, and were not necessarily part of traditional dishes. Traditional dishes were considered healthy and sustainable in this sample and are mostly based on cooked cereals (e.g. millet, sorghum, rice, maize) processed to tô, as well as on beans and leaves, often with a sauce [40–42]. Furthermore, it is important to note, that meat did not play a significant role in our interviews, possibly because meat consumption of this Burkinabé population group is low in general [12,40]. The EAT-Lancet PHD acknowledges that meat consumption is low in SSA and that an adequate intake of animal-based foods can improve nutritional status and health in populations were under- and malnutrition are prevalent [20].

Finally, taste and enjoyment were described as features of healthy eating in the literature [36,39,43] but not so much in our sample. This underrepresented perspective could be explained by a focus on sufficient food for the family and frequent experiences of hunger. A qualitative study with rural Ethiopian children also noted this priority of satisfying hunger [44].

While quantitative literature positions Burkina Faso in the early stages of the nutrition transition [9], the participants in the present study already experience changes in the food system, as well as in their dietary practices and their children’s preferences. This might be due to the fact that the experienced transitions rather took place within food groups and referred to the replacement of one cereal for another (e.g. rice for tô) or one production process for another (e.g. organic/home-made vs. pesticide-use/bought). Such relevant transitions are not easily detectable with traditional quantitative assessment instruments, like food frequency questionnaires. Additionally, the above-mentioned inter-generational tensions regarding dietary preferences can be interpreted as another indicator of the nutrition transition.

### Implications for public health action to promote healthy and environmentally sustainable diets

In our sample, many participants wished to learn about the interactions between nutrition, health, and the environment. This can be evaluated as beneficial for the acceptability of information campaigns to enhance people’s understanding of the environmental impacts of nutrition and to empower self-determined choices. This is also being proposed in the ‘Determinants Of Nutrition And Eating’ framework [45]. Ideally, such campaigns would align with national dietary guidelines. A review published in 2022 [46] highlighted that dietary guidelines, including those for Burkina Faso, often lack recommendations for sustainable diets, indicating a need for revisions.

As traditional Burkinabé diets usually contain less sugar, fat, and salt, and more cereals and legumes than modern dishes [47], public health interventions should build on the existing perception that such traditional diets are healthy and environmentally sustainable. Simultaneously, nutrient deficiencies remain a public health challenge [17,47], which is why dietary diversity needs to be enhanced in Burkina Faso [47]. While focusing on the promotion of plant-based diets, the intake of animal-based foods in accordance with the EAT-Lancet Commission’s recommendations could also be encouraged by dietary interventions in this context [20]. Moreover, there is evidence that vegetable consumption in SSA, particularly among individuals with lower socio-economic status, remains insufficient and strongly contributes to the development of NCDs and all-cause mortality [48]. Climate change could further reduce the availability of fruits and vegetables until 2050 and aggravate this problem [49]. Several participants shared observations of a changing climate in Ouagadougou, which are supported by an analysis of the World Meteorological Organization that predicts an outstanding impact of climate change on the African continent [50]. As some of the respondents expressed the wish to cultivate their own food, so-called home- or micro-gardens could be an option to promote the urban populations’ fruit and vegetable consumption and enhance household food security [51]. To evaluate the acceptability, feasibility, and effectiveness of home-gardening in urban Burkina Faso, more research is required.

Economic barriers were a major barrier to eat healthy and environmentally sustainable according to our participants. This resonates with the Food and Agricultural Organization’s concept of sustainable diets, where economic affordability is an integral dimension [52]. Potential approaches to achieve affordability are the implementation of subsidies on healthy foods or the introduction of taxes on unhealthy food [53–55]. If the tax revenues were used to strengthen the health system and the food system, health equity could be improved [5,55,56]. The recent implementation of a sugar-sweetened beverage tax in Nigeria [57] could serve as an example for similar efforts in other Western SSA countries, including Burkina Faso.

Moreover, since insufficient availability of healthy and environmentally sustainable foods at the workplace has been identified as another barrier, a facilitating environment needs to be promoted. In the literature, a healthy work environment has been identified to be key for preventing nutrition-related NCDs [58]. This is becoming increasingly important in the changing employment landscape in urban Burkina Faso, leaving less time for food preparation according to the respondents.

Finally, participants reported on climate change impairing local food production. Therefore, support of local small-scale farmers to increase their climate-resilience is necessary [59,60], ideally embedded in a global food strategy [61].

### Strengths and limitations

This study is the first that explored in-depth perceptions of healthy and environmentally sustainable diets and associated barriers in a sample of adults living in different settlement types of urban Burkina Faso. A strength of the study is the structured recruitment through the Ouagadougou HDSS including stratification for sex and settlement type. We followed principles of trustworthiness in qualitative research as described in additional file 3 (SRQR checklist) [62,63]. A limitation of this study included the COVID pandemic, preventing HF and AH from travelling to Burkina Faso for data collection and contextual learning. However, the contributions from researchers in Burkina Faso, namely SZ and RMM, facilitated the incorporation of local perspectives into the analysis, interpretation, and writing process.

## Conclusion

Quantitative studies place the population of Ouagadougou in early stages of the nutrition transition. In contrast, our study participants clearly saw changes in their diet and altering preferences towards ‘modern’ diets in the young generation. As children and adolescents were not part of this study, more research is needed on this generations’ food preferences to determine if they align with the results from the present study. Except for low dietary diversity, adult participants viewed their traditional diet as healthy and environmentally sustainable. As many components of traditional diets are in line with evidence-based recommendations on sustainable and healthy eating, this can be seen as an asset for public health programs focusing on the implementation of healthy and environmentally sustainable diets. Such programs should empower people to overcome perceived barriers like limited food affordability and availability with a focus on educational programs, supporting local and climate-resilient food production, as well as regulative measures to increase affordability of healthy foods.

## Supplementary Material

AdditionalFile3SRQRchecklist.docx

AdditionalFile1SubcategoriesCategoriesCore theme.docx

AdditionalFile2QuotesEnFr.docx

## Data Availability

The dataset generated and analysed during the current study is not publicly available due to privacy considerations but may be available from the corresponding author on reasonable request.

## List of abbreviations

Health and Demographic Surveillance System: HDSS Ouagadougou
Non-communicable diseases: NCD
Planetary Health Diet: PHD
Sub-Saharan Africa: SSA
Quotes
Px: Pseudonymized participant ID
Sex: m: male/f=female
Settlement type: fo: formal/if=informal

## References

[1] Popkin BM, Adair LS, Ng SW. Global nutrition transition and the pandemic of obesity in developing countries. Nutr Rev. 2012;70:3–14. doi: 10.1111/j.1753-4887.2011.00456.x Cited in: PubMed; PMID 22221213. 22221213 PMC3257829

[2] Steyn NP, McHiza ZJ. Obesity and the nutrition transition in Sub-Saharan Africa. Ann N Y Acad Sci. 2014;1311:88–101. doi: 10.1111/nyas.12433 Cited in: PubMed; PMID 24725148. 24725148

[3] Ford ND, Patel SA, Narayan KMV. Obesity in low- and middle-income countries: burden, drivers, and emerging challenges. Annu Rev Public Health. 2017:38145–38164. doi: 10.1146/annurev-publhealth-031816-044604 Cited in: PubMed; PMID 28068485. 28068485

[4] Popkin BM, Gordon-Larsen P. The nutrition transition: worldwide obesity dynamics and their determinants. Int J Obes Relat Metab Disord. 2004;28:S2–S9. doi: 10.1038/sj.ijo.0802804 Cited in: PubMed; PMID 15543214. 15543214

[5] Popkin BM, Ng SW. The nutrition transition to a stage of high obesity and noncommunicable disease prevalence dominated by ultra-processed foods is not inevitable. Obes Rev. 2022;23:e13366. doi: 10.1111/obr.13366 Cited in: PubMed; PMID 34632692. 34632692 PMC8639733

[6] Abrahams Z, McHiza Z, Steyn NP. Diet and mortality rates in Sub-Saharan Africa: stages in the nutrition transition. BMC Public Health. 2011;11:11801. doi: 10.1186/1471-2458-11-801 Cited in: PubMed; PMID 21995618. PMC3209469 21995618

[7] Benziger CP, Roth GA, Moran AE. The global burden of disease study and the preventable burden of NCD. Glob Heart. 2016;11:393–397. doi: 10.1016/j.gheart.2016.10.024 Cited in: PubMed; PMID 27938824. 27938824

[8] Gouda HN, Charlson F, Sorsdahl K, et al. Burden of non-communicable diseases in Sub-Saharan Africa, 1990–2017: results from the global burden of disease study 2017. Lancet Global Health. 2019;7:e1375–e1387. doi: 10.1016/S2214-109X(19)30374-2 Cited in: PubMed; PMID 31537368. 31537368

[9] Cisse K, Samadoulougou S, Ouedraogo M, et al. Prevalence of abdominal obesity and its association with cardiovascular risk among the adult population in Burkina Faso: findings from a nationwide cross-sectional study. BMJ Open. 2021;11:1–12. doi: 10.1136/bmjopen-2021-049496 Cited in: PubMed; PMID 34230021. PMC8261883 34230021

[10] Country Profile Burkina Faso [Internet]. 2021 [updated 2021 Aug 20; cited 2022 Jun 22]. Available from: https://ncdrisc.org/country-profile.html

[11] Casari S, Di Paola M, Banci E, et al. Changing dietary habits: the impact of urbanization and rising socio-economic status in families from Burkina Faso in Sub-Saharan Africa. Nutrients. 2022;14. doi: 10.3390/nu14091782 Cited in: PubMed; PMID 35565752. PMC9104313 35565752

[12] Weil K, Coulibaly I, Fuelbert H, et al. Dietary patterns and their socioeconomic factors of adherence among adults in urban Burkina Faso: a cross-sectional study. J Health Popul Nutr. 2023;42:107. doi: 10.1186/s41043-023-00451-w Cited in: PubMed; PMID 37817202. 37817202 PMC10566033

[13] Zeba AN, Delisle HF, Renier G. Dietary patterns and physical inactivity, two contributing factors to the double burden of malnutrition among adults in Burkina Faso, West Africa. J Nutr Sci. 2014:31–14. doi: 10.1017/jns.2014.11 Cited in: PubMed; PMID 26101618. PMC4473138 26101618

[14] Assmus F, Galbete C, Knueppel S, et al. Carbohydrate-dense snacks are a key feature of the nutrition transition among Ghanaian adults - findings from the RODAM study. Food Nutr Res. 2021;65. doi: 10.29219/fnr.v65.5435 Cited in: PubMed; PMID 34512231. PMC8388941 34512231

[15] Rossier C, Soura AB, Duthé G, et al. Non-communicable disease mortality and risk factors in formal and informal neighborhoods, Ouagadougou, Burkina Faso: evidence from a health and demographic surveillance system. PLOS ONE. 2014;9:1–18. doi: 10.1371/journal.pone.0113780 Cited in: PubMed; PMID 25493649. PMC4262303 25493649

[16] Diendéré J, Kaboré J, Somé JW, et al. Prevalence and factors associated with overweight and obesity among rural and urban women in Burkina Faso. Pan Afr Med J. 2019;34:34199–34211. doi: 10.11604/pamj.2019.34.199.20250 Cited in: PubMed; PMID 32180873. PMC7060945 32180873

[17] Zeba AN, Delisle HF, Renier G, et al. The double burden of malnutrition and cardiometabolic risk widens the gender and socio-economic health gap: a study among adults in Burkina Faso (West Africa). Public Health Nutr. 2012;15:2210–2219. doi: 10.1017/S1368980012000729 Cited in: PubMed; PMID 22463806. 22463806 PMC10271501

[18] Clark MA, Springmann M, Hill J, et al. Multiple health and environmental impacts of foods. Proc Natl Acad Sci USA (PNAS). 2019;116:23357–23362. doi: 10.1073/pnas.1906908116 Cited in: PubMed; PMID 31659030. 31659030 PMC6859310

[19] Clark MA, Domingo NGG, Colgan K, et al. Global food system emissions could preclude achieving the 1.5° and 2°C climate change targets. Science. 2020;370:705–708. doi: 10.1126/science.aba7357 Cited in: PubMed; PMID 33154139. 33154139

[20] Willett W, Rockström J, Loken B, et al. Food in the Anthropocene: the eat–lancet commission on healthy diets from sustainable food systems. Lancet. 2019;393:447–492. doi: 10.1016/S0140-6736(18)31788-4 30660336

[21] Sampson UKA, Amuyunzu-Nyamongo M, Mensah GA. Health promotion and cardiovascular disease prevention in Sub-Saharan Africa. Prog Cardiovasc Dis. 2013;56:344–355. doi: 10.1016/j.pcad.2013.10.007 Cited in: PubMed; PMID 24267442. 24267442

[22] Hardy-Johnson P, Dhuria P, Strommer S, et al. Exploring the diet and physical activity behaviours of adolescents living in India and sub-Saharan Africa: a qualitative evidence synthesis. Public Health Nutr. 2021;24:5288–5298. doi: 10.1017/S1368980021002408 Cited in: PubMed; PMID 34196267. 34196267 PMC10201354

[23] Hoek AC, Pearson D, James SW, et al. Shrinking the food-print: a qualitative study into consumer perceptions, experiences and attitudes towards healthy and environmentally friendly food behaviours. Appetite. 2017;108:117–131. doi: 10.1016/j.appet.2016.09.030 Cited in: PubMed; PMID 27686818. 27686818

[24] Paquette M-C. Perceptions of healthy eating: state of knowledge and research gaps. Can J Public Health. 2005;96:S16–21. Cited in: PubMed; PMID 16042159. doi: 10.1007/BF0340519616042159

[25] Pinto VRA, Campos R, Rocha F, et al. Perceived healthiness of foods: a systematic review of qualitative studies. Future Foods. 2021;4:4100056. doi: 10.1016/j.fufo.2021.100056

[26] Osei-Kwasi H, Mohindra A, Booth A, et al. Factors influencing dietary behaviours in urban food environments in Africa: a systematic mapping review. Public Health Nutr. 2020;23:2584–2601. doi: 10.1017/S1368980019005305 Cited in: PubMed; PMID 32450938. 32450938 PMC7116038

[27] Yu E, Malik VS, Hu FB. Cardiovascular disease prevention by diet modification. JACC Health Promot Ser J Am Coll Cardiol. 2018;72:914–926. doi: 10.1016/j.jacc.2018.02.085 Cited in: PubMed; PMID 30115231. PMC6100800 30115231

[28] Herrmann A, Gonnet A, Millogo RM, et al. Sustainable dietary weight loss intervention and its effects on cardiometabolic parameters and greenhouse gas emissions: study protocol of a randomised controlled trial with overweight and obese adults in Ouagadougou, Burkina Faso. BMJ Open. 2023;13:e070524. doi: 10.1136/bmjopen-2022-070524 Cited in: PubMed; PMID 37015795. PMC10083789 37015795

[29] Rossier C, Soura A, Baya B, et al. Profile: the Ouagadougou health and demographic surveillance system. Int J Epidemiol. 2012;41:658–666. doi: 10.1093/ije/dys090 Cited in: PubMed; PMID 22685112. 22685112 PMC3396324

[30] UNDP. Burkina Faso [Internet]. 2024 [cited 2024 Dec 7; cited 2024 Dec 7]. Available from: https://data.undp.org/countries-and-territories/BFA

[31] Moser A, Korstjens I. Series: practical guidance to qualitative research. Part 3: sampling, data collection and analysis. Eur J Gener Pract. 2018;24:9–18. doi: 10.1080/13814788.2017.1375091 PMC5774281 29199486

[32] Low J. Unstructured and semi-structured interviews in health research. In: Saks M, and Allsop J, editors. Researching health, qualitative, quantitative and mixed methods. Los Angeles, Calif: SAGE Publications; 2013. p. 123–141.

[33] Braun V, Clarke V. Using thematic analysis in psychology. Qualitative Res Phychol. 2006;3:77–101. doi: 10.1191/1478088706qp063oa

[34] Cypress BS. Qualitative research: the “what,” “why,” “who,” and “how”! Dimensions of critical care nursing. 2015;34:356–361. doi: 10.1097/DCC.0000000000000150 Cited in: PubMed; PMID 26436302. 26436302

[35] Moriarty J. Qualitative methods: overview. School for Social Care Research. London: School of Economics and Political Science, National Institute for Health Research; 2011. p. 1–43.

[36] Bisogni CA, Jastran M, Seligson M, et al. How people interpret healthy eating: contributions of qualitative research. J Nutr Educ Behav. 2012;44:282–301. doi: 10.1016/j.jneb.2011.11.009 Cited in: PubMed; PMID 22732708. 22732708

[37] Hazley D, Stack M, Kearney JM. Perceptions of healthy and sustainable eating: a qualitative study of Irish adults. Appetite. 2024;192:107096. doi: 10.1016/j.appet.2023.107096 Cited in: PubMed; PMID 37890530. 37890530

[38] Herrmann A, Sauerborn R, Nilsson M. The role of health in households’ balancing act for lifestyles compatible with the Paris agreement—qualitative results from Mannheim, Germany. Int J Environ Res Public Health. 2020;17:1297. doi: 10.3390/ijerph17041297 Cited in: PubMed; PMID 32085458. 32085458 PMC7068404

[39] Iradukunda F, Poudel-Tandukar K. Healthy diet perceptions of pregnant women from sub-saharan Africa residing in the U.S. Ecol Food Nutr. 2021;60:682–696. doi: 10.1080/03670244.2021.1875457 Cited in: PubMed; PMID 33467928. 33467928

[40] Becquey E, Savy M, Danel P, et al. Dietary patterns of adults living in Ouagadougou and their association with overweight. Nutr J. 2010;9:91–10. doi: 10.1186/1475-2891-9-13 Cited in: PubMed; PMID 20307296. PMC2848625 20307296

[41] Dabo R, Hama-Ba F, Savadogo A. Food typology of traditional foods based on millet, sorghum and cowpea from the rural communes of north central region of Burkina Faso. Food Nutr Sci. 2023;15:681–694. doi: 10.4236/fns.2024.158043

[42] Aminata G. Food habits and nutrition security in West Africa: practices from Southwestern Burkina Faso [dissertation]. Universität Bonn. Bonn; 2020. [cited 2024 Dec 7.

[43] Tobler C, Visschers VHM, Siegrist M. Eating green. Consumers’ willingness to adopt ecological food consumption behaviors. Appetite. 2011;57:674–682. doi: 10.1016/j.appet.2011.08.010 Cited in: PubMed; PMID 21896294. 21896294

[44] Morrow V, Tafere Y, Chuta N, et al. “I started working because I was hungry”: the consequences of food insecurity for children’s well-being in rural Ethiopia. Social Science & Medicine. 2017;182:1–9. doi: 10.1016/j.socscimed.2017.04.004 Cited in: PubMed; PMID 28407566. 28407566

[45] Stok FM, Hoffmann S, Volkert D, et al. The DONE framework: creation, evaluation, and updating of an interdisciplinary, dynamic framework 2.0 of determinants of nutrition and eating. PLOS ONE. 2017;12:e0171077. doi: 10.1371/journal.pone.0171077 Cited in: PubMed; PMID 28152005. 28152005 PMC5289713

[46] Ainuson-Quampah J, Amuna NN, Holdsworth M, et al. A review of food-based dietary guidelines in Africa: opportunities to enhance the healthiness and environmental sustainability of population diets. AJFAND. 2022;22:19471–19495. doi: 10.18697/ajfand.107.21790

[47] Nikiema L, Sawadogo SP, Lanou H, et al. Pratiques d’alimentation des ménages au Burkina Faso, sources des apports journaliers totaux en énergie, macronutriments et micronutriments/Feeding practices of households in Burkina Faso, source of daily total energy, macronutrient, and micronutrients intake. SS [Internet]. 2010;33. Available from: https://revuescience-techniqueburkina.org/index.php/sciences_de_la_sante/article/view/512

[48] Frank SM, Webster J, McKenzie B, et al. Consumption of fruits and vegetables among individuals 15 years and older in 28 low- and middle-income countries. J Nutr. 2019;149:1252–1259. doi: 10.1093/jn/nxz040 Cited in: PubMed; PMID 31152660. 31152660

[49] Springmann M, Mason-D’Croz D, Robinson S, et al. Global and regional health effects of future food production under climate change: a modelling study. Lancet. 2016;387:1937–1946. doi: 10.1016/S0140-6736(15)01156-3 26947322

[50] World Meteorological Organization. Africa faces disproportionate burden from climate change and adaptation costs [Internet]. 2024 [cited 2024 Nov 13; cited 2024 Dec 7]. Available from: https://wmo.int/media/news/africa-faces-disproportionate-burden-from-climate-change-and-adaptation-costs

[51] Marsh R. Building on traditional gardening to improve household food security. Food Nutr Agric [Internet]. 1998:4–14. Available from: https://www.semanticscholar.org/paper/Building-on-traditional-gardening-to-improve-food-Marsh/ce011b6bcd0326ac9cb5d4c5f262eb45dd9231ff

[52] Barbara Burlingame, Sandro Dernini, FAO, editors. Sustainable diets and biodiversity: directions and solutions for policy, research and action. Rome, Italy: Food and Agriculture Organization of the United Nations; 2012 [cited 2021 Nov 1].

[53] Kenny TA, Woodside JV, Perry IJ, et al. Consumer attitudes and behaviors toward more sustainable diets: a scoping review. Nutr Rev. 2023;81:1665–1679. doi: 10.1093/nutrit/nuad033 Cited in: PubMed; PMID 37014671. 37014671 PMC10639109

[54] McGill R, Anwar E, Orton L, et al. Are interventions to promote healthy eating equally effective for all? Systematic review of socioeconomic inequalities in impact. BMC Public Health. 2015;15. doi: 10.1186/s12889-015-1781-7 Cited in: PubMed; PMID 25934496. PMC4423493 25934496

[55] Niebylski ML, Redburn KA, Duhaney T, et al. Healthy food subsidies and unhealthy food taxation: a systematic review of the evidence. Nutrition. 2015;31:787–795. doi: 10.1016/j.nut.2014.12.010 Cited in: PubMed; PMID 25933484. 25933484

[56] Backholer K, Sarink D, Beauchamp A, et al. The impact of a tax on sugar-sweetened beverages according to socio-economic position: a systematic review of the evidence. Public Health Nutr. 2016;19:3070–3084. doi: 10.1017/S136898001600104X Cited in: PubMed; PMID 27182835. 27182835 PMC10270974

[57] NCD Alliance. Nigeria sugary drinks tax aims to fight obesity, raise revenue [Internet]. 2022 [updated 2022 Mar 1; cited 2022 Nov 7]. Available from: https://ncdalliance.org/news-events/news/nigeria-sugary-drinks-tax-aims-to-fight-obesity-raise-revenue?utm_source=linkedin&utm_medium=website&utm_campaign=nigeria_sugary_drinks

[58] Mensink F, Schwinghammer SA, Smeets A. The healthy school canteen programme: a promising intervention to make the school food environment healthier. J Environ Public Health. 2012;2012:1–8. doi: 10.1155/2012/415746 Cited in: PubMed; PMID 22690228. PMC3368483 22690228

[59] Fan S, Rue C. The role of smallholder farms in a changing world. In: Gómez Y, Paloma S, Riesgo L Louhichi K, editors. The role of smallholder farms in food and nutrition security. Cham: Springer Nature; 2020. p. 13–28. (Springer eBook Collection).

[60] Sorgho R, Mank I, Kagoné M, et al. “We will always ask ourselves the question of how to feed the family”: subsistence farmers’ perceptions on adaptation to climate change in Burkina Faso. Int J Environ Res Public Health. 2020;17. doi: 10.3390/ijerph17197200 Cited in: PubMed; PMID 33019715. PMC7579300 33019715

[61] Kashwan P. Climate justice in the global north. Case Stud Environ. 2021;5:1–13. doi: 10.1525/cse.2021.1125003

[62] Cope DG. Methods and meanings: credibility and trustworthiness of qualitative research. Oncol Nurs Forum. 2014;41:89–91. doi: 10.1188/14.ONF.89-91 Cited in: PubMed; PMID 24368242. 24368242

[63] Nowell LS, Norris JM, White DE, et al. Thematic analysis: striving to meet the trustworthiness criteria. Int J Qualitative Methods. 2017;16:1–13. doi: 10.1177/1609406917733847

