# Supplementary material for: Concepts of healthy and environmentally sustainable diets clash with a life in transition – Findings from a qualitative study in urban Burkina Faso
Source: Glob Health Action. 2025 Feb 12;18(1):2457193. doi: 10.1080/16549716.2025.2457193 (PMC11823391; doi:10.1080/16549716.2025.2457193)
Supplement: AdditionalFile3SRQRchecklist.docx [file ZGHA_A_2457193_SM2071.docx]

SRQR Reporting checklist for qualitative study ^[[1]](#footnote-1)^

**Page(s)** [submitted manuscript]

**Title** 1

**Abstract** see ‘abstract’

**Introduction**

Problem formulation: 2-3

Purpose or research question: 3

**Methods**

Qualitative approach/research paradigm: 3-6

Researcher characteristics and reflexivity: 6

Context: 4 (+ Figure 1)

Sampling strategy: 4

Ethical issues: 28

Data collection methods: 4-5

Data collection instruments and technologies: 4-5 (+ Table 1)

Units of study: 7

Data processing: 5

Data analysis: 5-6

Techniques to enhance trustworthiness: 5, 6, 17

**Results**

Syntheses and interpretation: 6-16 (+ Figure 2; Additional file 1)

Links to empirical data: *Single quotes*: p.11; p.16 *Tables No. 2-12*;

*Additional file 2*

**Discussion**

Integration with prior work, implications,

transferability and contribution(s) to the field: 16-20

Limitations: 17

**Other**

Conflicts of interest: 28

Funding: 28-29

1. SRQR reporting guidelines: O'Brien BC, Harris IB, Beckman TJ, Reed DA, Cook DA. Standards for reporting qualitative research: a synthesis of recommendations. Acad Med. 2014;89(9):1245-1251. [↑](#footnote-ref-1)
