# Supplementary material for: Concepts of healthy and environmentally sustainable diets clash with a life in transition – Findings from a qualitative study in urban Burkina Faso
Source: Glob Health Action. 2025 Feb 12;18(1):2457193. doi: 10.1080/16549716.2025.2457193 (PMC11823391; doi:10.1080/16549716.2025.2457193)
Supplement: AdditionalFile1SubcategoriesCategoriesCore theme.docx [file ZGHA_A_2457193_SM2070.docx]

*Additional file 1*: Overview of subcategories, categories, and the core theme.

| Subcategory | Category | Core Theme |
| --- | --- | --- |
| Traditional | Characteristics of ideal healthy  and environmentally  friendly diets | Ideals of healthy and  environmentally friendly diets  clash with life in transition |
| Local |  |  |
| Transparent |  |  |
| Natural & Pure |  |  |
| Organic |  |  |
| Further features of healthy diets |  |  |
| Financial Resources | Barriers to implement healthy and  environmentally friendly diets |  |
| Availability & Offer |  |  |
| Time & Feasibility |  |  |
| Others, especially family |  |  |
| Lack of knowledge |  |  |
| Globalization of the  food system | Current transitions experienced  by the interviewees |  |
| Modern lifestyles,  Working for a living |  |  |
| Environmental change &  Increasing pollution |  |  |
