# Supplementary material for: Concepts of healthy and environmentally sustainable diets clash with a life in transition – Findings from a qualitative study in urban Burkina Faso
Source: Glob Health Action. 2025 Feb 12;18(1):2457193. doi: 10.1080/16549716.2025.2457193 (PMC11823391; doi:10.1080/16549716.2025.2457193)
Supplement: AdditionalFile2QuotesEnFr.docx [file ZGHA_A_2457193_SM2069.docx]

*Additional File 2*: Overview of all quotes, including the original French quotes.

|  | **Participant** | **Original quote** | **English translation** |
| --- | --- | --- | --- |
| Table 2 | P28, m, fo, 41 years | « Nos grands-parents consommaient ce qu’ils cultivaient. Ils connaissaient la provenance de leurs produits et même la sauce-là. Ce que les femmes prenaient c’était ce qu’ils cultivaient. Donc tout était vraiment naturel. […] Les qualités des sauces aussi ne sont pas pareilles. Les grands-parents faisaient leurs sauces et écrasaient leurs arachides pour préparer la pâte d’arachide mais aujourd’hui on achète tout. » | “Our grandparents consumed what they grew. They knew where their products came from and even the sauce. What the women used was what they grew. So everything was truly natural. [...] The quality of the sauces is different as well. The grandparents made their sauces and crushed their peanuts to make peanut paste, but today we buy everything” |
|  | P22, m, fo, 33 years | « Je me dis que nos grand parents, leur nourriture était beaucoup saine, c’était préparé de manière plus simple avec le soumbala qu’on gagnait et puis c’était du soumbala de bonne qualité » | “I tell myself that our grandparents, their food was much healthier, it was prepared in a simpler way with the soumbala that they gained and apart from that, it was good quality soumbala” |
|  | P15, f, if, 30 years | « Pour moi la différence c’est qu’au temps de nos grand-parents, il n’y avait pas de variété de plats. C’était le tô ou encore le haricot un truc comme ça, Mais actuellement on peut faire beaucoup de plats, donc on peut varier. [...] On n’avait pas le choix. » | "For me, the difference is that in our grandparents' time, there was no variety of dishes. It was either tô or beans, something like that. But nowadays, we can make a lot of dishes, so we can vary. [...] They didn't have a choice.” |
|  | P28, m, fo, 41 years | « Je pense que l’alimentation traditionnelle bien préparé est saine et durable. » | “I think that well-prepared traditional food is healthy  and sustainable.” |
| Table 3 | P28, m, fo, 41 years | « Mais si tu sais que le riz que tu as acheté était cultivé dans tel village et puis tu peux attester de tout son processus jusqu’à ça vient chez toi, bon là on disait que c’est authentique et ça va. » | “But if you know that the rice you bought was grown in that village and then you can confirm its entire process up to the point where it comes to you, well then we would say that it’s authentic and that it's fine.” |
|  | P3, m, fo, 60 years | « Ce que j’allais dire là, ce que moi je consomme là, le riz qu’on produit ici là ça c’est vraiment sain et durable. Ça me plait et je sais que ça me fait pas mal comme ça ! » | “What I was going to say here, what I consume, the rice that we produce here is really healthy and sustainable. I like it and I know that it doesn't inflict anything bad like that!” |
|  | P17, f, fo, 32 years | « Bon mes plats préférés, ce sont les plats africains hein, c’est surtout les mets locaux, le babenda surtout, […] la salade et puis l’attiéké, le riz […] j’aime ces plats parce que je me dis qu’au moins ils sont des mets qui sont bon à 80% c’est naturel et puis c’est du bio » | “My favourite dishes are the African ones, especially the local ones: Babenda, [...] salad and attiéké, rice [...] I like these dishes because I think that they are at least 80% good, they are natural and they are organic as well” |
| Table 4 | P15, f, if, 30 years | « Pour moi [une conduite alimentaire saine qui respecte l’environnement] c’est insister sur les produits naturels. Les produits qui ne sont pas passés par les usines. » | “For me [a healthy diet that respects the environment] is to insist on natural products. Products that have not  gone through factories.” |
| Table 5 | P28, m, fo, 41 years | « […] il y a les pesticides et tout ça qu’on utilise pour produire nos aliments. Alors que ces pesticides et autres polluent aussi le sol. Donc ça peut entrainer le changement climatique.“ | “[…] there are pesticides and so on that we use to produce our food. But these pesticides and others also pollute the soil. So that can drive climate change.” |
|  | P22, m, fo, 33 years | « Plus c’est loin et que le temps dure on sera obligé d’utiliser des produits chimiques pour pouvoir conserver. » | “The further away it is and the longer it takes, the more we will be forced to use chemicals to preserve it.” |
|  | P15, f, if, 30 years | « […] il y a des aliments qui sont traités à parti des usines. On utilise des machines qui polluent l’air. » | “[…] there are foods that are processed in factories. Machines are used that pollute the air.” |
| Table 6 | P3, m, fo, 60 years | « La famille, si tu fais manger ta famille correctement, c’est le riz local et bio, c’est sain. » | “The family, if you feed your family properly, it's local and organic rice, that's healthy.” |
|  | P28, m, fo, 41 years | « Sinon, si on mange essentiellement du bio, je pense que ça peut résoudre un peu ce phénomène [du changement climatique]. » | “Otherwise, if you eat mainly organic food, I think that this can solve the phenomenon [of climate change] a bit.” |
| Table 7 | P34, m, if, 49 years | « Des aliments sains […] on sait c’est que c’est issu de telle ou telle chose, ce qu’on a mis dans notre alimentation » | “Healthy food [...] we know that it is made from this or that thing, what we have put in our food” |
|  | P22, m, fo, 33 years | « Les aliments malsains c’est la consommation des produits vraiment qu’on ne sait pas d’où ça vient et dans quelle conditions ces produits ont été fabriqués ; c’est ce que j’appelle produits malsains. » | “Unhealthy food is the consumption of products of which you really don't know where they come from and under what conditions they were made; that’s what I call unhealthy products.” |
| Table 8 | P23, f, if, 50 years | « Si tu vas faire ta cuisine, il faut respecter l’hygiène. Tu ne dois pas faire la cuisine dans la saleté. […] Si la nourriture est faite dans les conditions non hygiéniques ça peut provoquer des maladies. Si c’est bien fait, quand on va la manger nous serons en bonne santé. » | “If you are cooking, you must respect hygiene. You must not cook in the dirt. [...] If food is cooked in unhygienic conditions, diseases can be provoked. If it is well done, when we eat it, we will be healthy.” |
|  | P7, f, fo, 31 years | « Si tu me vois manger au dehors donc c’est mariage. Ou bien en tout cas une cérémonie. Sinon je ne paies pas pour manger. […] C’est à cause de l’hygiène que je fais ça. Parce que je ne peux pas contrôler tout ce que les gens font. Tu pars payer tu viens manger, tu as des problèmes après. Tu as pris 200f pour aller payer maladie. Tu vas venir, comment dirais-je, soigner plus que tes 200f. Donc je préfère me garder chez moi pour faire ce que je peux pour manger. » | “If you see me eating outside, it's a wedding. Or at least a ceremony. Otherwise, I don't pay to eat. [...] I do this because of hygiene. Because I can't control everything that people do. You leave to pay, you come to eat, you have problems afterwards. You took 200f to go out and pay illness. You're going to come, how shall I say, to treat more than your 200f. So I prefer to keep myself at home to do what I can to eat.” |
|  | P14, f, fo, 50 years | « Nous aimerions par exemple varier nos plats. Manger le riz à midi, le tô le soir et pourquoi pas de l’igname le lendemain ce serait beaucoup de vitamines. Sinon si on se contente du tô l’organisme ne reçoit pas de vitamine. Le tô ne contient rien.» | “We would like to vary our dishes for example. Eating rice at noon, tô in the evening and why not yam the next day, that would be a lot of vitamins. Otherwise, if we content ourselves with the tô, the body does not receive any vitamins. The tô contains nothing.” |
|  | P16, m, fo, 48 years | « Bon, il y’a quand même la question de la qualité de ce qu’on veut manger. Même si on mange pour donner de l’énergie au corps on a quand même bien envie de ne pas souffrir après pour cela. » | “Well, there is also the question of quality of what we like to eat. Even if we eat to give energy to the body, we still don’t want to suffer afterwards.” |
|  | P15, f, if, 30 years | « Malsain pour moi c’est par exemple manger trop gras ou trop sucré. Trop de sel. » | "Unhealthy for me is for example eating too much fat or too much sugar. Too much salt." |
| Table 9 | P18, m, if, 55 years | « […] on est dans un monde qui est perdu, pour manger sain il faut avoir vraiment les moyens. Si tu n’as pas les moyens, tu ne peux pas bien manger voilà. » | “[…] we are in a world that is lost, to eat healthy you really have to have the means. If you don't have the means, you can't eat well. That's it." |
|  | P22, m, fo, 33 years | « […] dans ces boutiques vous partez vous regardez les différentes marques de riz, c’est un exemple hein, vous allez voir qu’il n y a pas de riz local. Pour avoir le riz local c’est difficile […] Donc ça c’est un obstacle. […] En plus de ça, je me dis que c’est très moins cher le riz importé par rapport au riz local, donc ces des difficultés. » | “[…] you go in these stores and look at the different brands of rice, this is an example, you will see that there is no local rice. It is difficult to get local rice [...] So that’s an obstacle. [...] In addition to that, I tell myself that imported rice is much cheaper than local rice, so these are difficulties.” |
|  | P13, m, if, 22 years | « Mais maintenant même souvent préparer à la maison, tout ça demande assez de moyens. Ce qui est déjà prêt, tu ne perds pas du temps, et ça ne demande pas aussi assez de moyens. » | “But now, cooking at home, it's quite expensive. What is already prepared, you don't waste time, and it doesn't require too many means either.” |
|  | P7, f, fo, 31 years | « Ce qui peut influencer la décision, ce sont les enfants. Si tu vas préparer et puis l’enfant ne va pas manger, ou bien ton mari n’aime pas tel repas, tu vas faire ce que la famille veut. […] ce que toi tu veux ça ne tient pas, si, tu fais pour les autres ! » | “The children can influence the decision. If you are going to cook and then the child doesn't eat, or your husband doesn't like the meal, you will do what the family wants. [...] what you want doesn't count, yes, you cook for the others!” |
|  | P34, m, if, 49 years | « Je viens de réaliser qu’au Burkina, même moi particulièrement on n’a pas une idée sur la nutrition. Quels sont les aliments qui peuvent apporter la santé à l’homme, quels sont les aliments que l’homme doit consommer pour avoir des vitamines ? La plupart et beaucoup ignorent cela. […] On n’a pas une idée quels aliments peut apporter quoi, quels aliments peut apporter tels vitamines. Bon c’est souvent quelques rare fois qu’on présente à la télé, mais on n’a pas des structures qui peuvent conseiller les gens à avoir des aliments sains. Donc ça fait que du coup l’ignorance aussi peut faire que beaucoup de gens vont consommer aussi croyant que ces de bons aliments alors que ce sont des aliments qui vont les tuer à petit feu. » | “I have just realized that in Burkina, even I, we don't have an idea about nutrition. What are the foods that can bring health to man, what are the foods that man must consume to have vitamins? Most do not know this. [...] We don't have an idea which foods can bring what, which foods provide vitamins. Well, some rare times they show it on TV, but we don't have structures that can advise people to have healthy food. So that's why ignorance can also make many people consume food believing that it is good food when in fact it is food that will kill them slowly.” |
| Table 10 | P28, m, fo, 41 years | « Il y a une grande différence  […] les habitudes alimentaires ont beaucoup changé. A l’époque c’était beaucoup plus le tô de mil, de maïs. Aujourd’hui c’est beaucoup plus le riz et puis les pâtes. Donc ce n’est pas la même chose. » | “There is a big difference [...] eating habits have changed a lot. In the past, it was a lot more tô made from millet, corn. Today it's much more rice and pasta. So it's not the same thing.” |
|  | P1, m, fo, 38 year | « Les plats traditionnels de nos jours sont devenus modernes maintenant. […] on a rendu ça moderne […] De nos jours si on veut préparer le haricot on le prépare avec des condiments on fait une sauce, on ajoute du maggi. Ce n’est plus du haricot ça devient autre chose. » | “Traditional dishes nowadays have become modern. [...] we have made them modern [...] Nowadays, if we want to prepare beans we prepare it with condiments, we make a sauce, we add Maggi. It's no longer beans, it becomes something else.” |
|  | P3, m, fo, 60 years | « […] l’engrais comme ça, produits chimiques là. Aujourd’hui là c’est ça seulement […] Et les choses ont changé parce qu’avant on le mangeait sans soucis car les repas n’étaient pas toxiques. Il n’y avait pas de produits chimiques. Mais aujourd’hui on ne consomme que du chimique. » | “[...] fertiliser like that, chemicals; today it's just that [...] And things have changed because in the past we used to eat without worries because the meals were not toxic. There were no chemicals. But today we only eat chemicals.” |
|  | P34, m, if, 49 years | « […] l’Europe n’était pas proche comme actuellement. Du jour au lendemain un riz peut quitter l’Amérique pour atterrir ici alors que nos grands parents ne connaissaient pas ça. » | “[…] Europe was not as close as it is today. From one day to the next rice can leave America and land here, whereas our grandparents did not know that.” |
|  | P23, f, if, 50 years | « […] les gens ont adopté ce qui est moderne au détriment de la nutrition de nos grands-parents. Et c’est ce qui fait que les maladies se multiplient. Le fait d’abandonner l’alimentation de nos grands-parents au profit de celle moderne c’est pourquoi on est exposé à plus de maladies. » | “[…] people have adopted what is modern at the expense of the nutrition of our grandparents. And that's why diseases are increasing. The fact that we are abandoning our grandparents' diet in favour of the modern one is why we are exposed to more diseases.” |
|  | P14, f, fo, 50 years | « Nos enfants passent leur journée le ventre vide parce qu’ils n’aiment pas le tô. Ils sont prêts à aller affamés à l’école à cause du tô. Mais quand on prépare le riz, ils sont alors tout contents. Mais quand c’est le tô, ils n’en mangent pas. Ils sont même prêts à dormir affamés. » | “Our children spend their day hungry because they don't like tô. They are ready to go to school hungry because of the tô. But when we prepare rice, they are very happy. But when it's tô, they don't eat it. They are even ready to sleep hungry.” |
| Table 11 | P16, m, fo, 48 years | « Les gens consacrent moins de temps pour se faire à manger qu’avant. Aujourd’hui par exemple, les femmes du fait qu’elles travaillent, elles ne sont pas toujours à la maison pour pouvoir prendre du temps pour préparer donc chacun cherche à faire rapide. Donc du coup ça amène à utiliser des produits manufacturés qui permet de faire vite et je crois que ça joue beaucoup sur le choix de l’alimentation. » | “People spend less time cooking than they used to. Today, for example, women, because they work, are not always at home to take their time to prepare food, so everyone is looking to prepare something quick. This leads to the use of manufactured products that allow you to do things quickly and I think that this plays a lot on food choices.” |
| Table 12 | P16, m, fo, 48 years | « […] avec l’augmentation des températures dès mars, on a moins d’eau. Donc, les maraichers ne peuvent plus continuer à produire dans leurs jardins. [...] Donc les prix vont rapidement s’augmenter [...] tu ne peux plus manger à base des produits frais. Voilà le lien au changement climatiques. Aussi, si la saison agricole a été très mauvaise dû aux irrégularités des pluies, il va s’en dire qu’on n’aura pas assez de maïs, du mil sur le marché et donc du coup on aura un problème de prix, et si les prix sont trop élevés on sera obligé de diminuer les quantités qu’on mange. Parlant de température aussi, […] parce qu’il fait beaucoup plus chaud, la demande de l’eau aussi augmente. » | “[…] with the increase in temperature from March, there is less water. So, the market gardeners can't continue to produce in their gardens. [...] So the prices will quickly increase [...] you can't eat fresh products anymore. This is the link to climate change. Also, if the agricultural season has been very bad due to irregular rainfall, it will mean that we won't have enough maize or millet on the market, and so we will have a price problem, and if the prices are too high we will be obliged to reduce the quantities that we eat. Speaking of the temperature too, [...] because it is much hotter, the demand for water is also increasing.” |
|  | P36, f, if, 47 years | « Même quand tu es malade on dit que c’est dû au changement de temps. » | "Even when you're sick we say that it's due to the weather change." |
|  | P34, m, if, 49 years | « Si on voit la prolifération des sachets, et même des bouteilles qu’on jette, tout ça, ça influence [...] ces sachets plastiques qui dégradent vraiment notre environnement, voilà tout ça et même la pollution [...] tout ça a des effets négatifs [...] c’est la dégradation, ça veut dire que ça détruit même le sol. » | “If we see the proliferation of bags, and even bottles that we throw away, all that, it has an impact [...] these plastic bags that really degrade our environment, that's it and the pollution [...] all that has negative effects [...] it's degradation, that means that it even destroys the soil.” |
| Single quotes embedded in the text | P18, m, if, 55 years | « Je suis un fonctionnaire, j’ai 31 ans de service mais je n’arrive pas à manger convenablement comme moi je veux. Moi je veux plutôt manger bio, tu vas gagner ça où aujourd’hui ? » | “I am a civil servant, I have 31 years of service but I can't eat properly as I want. I would like to eat organic, where are you going to get that today?” |
|  | P23, f, if, 50 years | « Du moment que ce n’est pas bon pour l’être humain, ils ne seront pas bons pour la terre également. » | “If it is not good for the human being, it is not good for the earth either.” |
|  | P7, f, fo, 31 years | « Pour se dire que moi je suis civilisé, il faut manger les repas de l’Europe. » | "civilized” |
|  | P2, f, fo, 53 years | « Il ne pleut plus assez, la chaleur est beaucoup plus qu’avant » | “It doesn't rain enough anymore, the heat is much stronger than before” |
|  | P25, f, fo, 42 years | « Dans toute chose c’est la santé qui compte. Si tu as la santé le reste tu peux faire. » | “In everything it is health that counts. If you are healthy, the rest you can do.” |
